# Neutralizing antibody and T cell responses against SARS-CoV-2 variants of concern following ChAdOx-1 or BNT162b2 boosting in the elderly previously immunized with CoronaVac vaccine

**DOI:** 10.1186/s12979-022-00279-8

**Published:** 2022-05-24

**Authors:** Chalerm Liwsrisakun, Supansa Pata, Witida Laopajon, Nuchjira Takheaw, Warawut Chaiwong, Juthamas Inchai, Chaicharn Pothirat, Chaiwat Bumroongkit, Athavudh Deesomchok, Theerakorn Theerakittikul, Atikun Limsukon, Pattraporn Tajarernmuang, Nutchanok Niyatiwatchanchai, Konlawij Trongtrakul, Kantinan Chuensirikulchai, Watchara Kasinrerk

**Affiliations:** 1 Division of Pulmonary, Critical Care, and Allergy, Department of Internal Medicine, Faculty of Medicine, Chiang Mai University, Chiang Mai, Thailand; 2 Division of Clinical Immunology, Department of Medical Technology, Faculty of Associated Medical Sciences, Chiang Mai University, Chiang Mai, Thailand; 3 Biomedical Technology Research Center, National Center for Genetic Engineering and Biotechnology, National Science and Technology Development Agency at the Faculty of Associated Medical Sciences, Chiang Mai University, Chiang Mai, Thailand

**Keywords:** SARS-CoV-2, Variants of concern, COVID-19, COVID-19 vaccine, Booster vaccine, Elderly

## Abstract

**Background:**

The existence of SARS-CoV-2 variants of concern (VOCs) in association with evidence of breakthrough infections despite vaccination resulted in the need for vaccine boosting. In elderly individuals, information on the immunogenicity of booster vaccinations is limited. In countries where the CoronaVac inactivated vaccine is the primary vaccine, the appropriate boosting regimen is not clear. Immunologic studies of the effects of booster vaccination against VOCs, particularly Delta and Omicron, following CoronaVac in elderly individuals are helpful for policy makers. In this study, we determined the immune responses against VOCs following ChAdOx-1 or BNT162b2 boosting in elderly individuals previously immunized with CoronaVac.

**Results:**

Before boosting, the median % inhibition of neutralizing antibodies (NAbs) against the wild-type (WT), Alpha, Beta, Delta and Omicron variants in the ChAdOx-1 and BNT162b2 groups was 52.8% vs. 53.4, 36.6% vs. 39.9, 5.2% vs. 13.7, 34.3% vs. 44.9, and 20.8% vs. 18.8%, respectively. After boosting with ChAdOx-1 or BNT162b2, the % inhibition of NAbs were increased to 97.3% vs. 97.4, 94.3% vs. 97.3%, 79.9 vs. 93.7, 95.5% vs. 97.5, and 26.9% vs. 31.9% for WT, Alpha, Beta, Delta and Omicron variants, respectively. Boosting with BNT162b2 induced significantly higher NAb levels than boosting with ChAdOx-1 against the Alpha, Beta and Delta variants but not the WT and Omicron variants. NAb levels against Omicron variant were not significantly different before and after boosting with ChAdOx-1 or BNT162b2. To evaluate T-cell responses, S peptides of the WT, Alpha, Beta and Delta variants were used to stimulate T cells. Upon stimulation, the expression of IL-17A in CD8 T cells was higher in the BNT162b2 group than in the ChAdOx-1 boosting group. However, IFN-γ production in CD4 and CD8 T cells did not significantly differ under all vaccination regimens. The expression of FasL in CD4 T cells, but not CD8 T cells, was higher in the BNT162b2-boosted group.

**Conclusion:**

Boosting with either ChAdOx-1 or BNT162b2 in CoronaVac-primed healthy elderly individuals induced high NAb production against all examined VOCs except Omicron. BNT162b2 stimulated higher NAb and some T-cell responses than ChAdOx-1. Vaccine boosting is, therefore, recommended for elderly individuals previously immunized with CoronaVac.

**Supplementary Information:**

The online version contains supplementary material available at 10.1186/s12979-022-00279-8.

## Introduction

COVID-19 has been causing a pandemic since early 2020. Several variants of concern (VOCs) have arisen from the ancestral Wuhan strain and become predominant circulating variants globally. Various COVID-19 vaccine platforms have been developed and authorized for emergency use worldwide. Vaccines have been proven to reduce disease severity and death [1]. However, mRNA vaccines have been reported to achieve the most effective prevention of SARS-CoV-2 infection for the first emerged variants [2]. Due to the unavailability of mRNA vaccines, the CoronaVac (inactivated) and ChAdOx-1 (virus-vectored) vaccines have been authorized for use as primary COVID-19 vaccines in Thailand since March 2021. Because health care workers (HCWs) are among the groups at highest risk of infection, they were the first group of the population to receive the first available CoronaVac in the country.

The standard 2-dose CoronaVac regimen can induce anti-SARS-CoV-2 receptor-binding domain (RBD) antibodies, neutralizing antibodies (NAbs) and cellular immunity in both the elderly and younger age groups [3, 4]. However, after the appearance of viral VOCs, particularly the Delta variant, there were reports of breakthrough infection among HCWs in Thailand and various other countries despite being fully vaccinated [5, 6]. Breakthrough infections caused by the Delta variant are associated with disease severity requiring hospitalization, especially in elderly individuals and patients with comorbidities [7]. The seroconversion rate of CoronaVac-induced NAbs against the Delta variant has been reported to be only 55.8% according to the plaque-reduction neutralization test [8]. Additionally, a study in Thailand showed that VOCs, particularly the Delta variant, are less susceptible to the effects of CoronaVac [9]. Therefore, for the safety of HCWs and their patients, the Thai Ministry of Public Health has developed a policy of boosting with either the ChAdOx-1 or BNT162b2 vaccine among HCWs previously immunized with 2 full doses of CoronaVac since July 2021. However, in November 2021, a new SARS-CoV-2 variant, the Omicron variant, was reported by the World Health Organization (WHO) [10] and quickly became the globally dominant variant. The emergence of the Omicron variant raised serious concerns due to the potential for immune escape from vaccine-induced humoral immunity [11]. The primary 2-dose series of the BNT162b2 mRNA vaccine was shown to be insufficient to protect against Omicron variant infection, and a third booster dose was suggested [11].

Ageing is one of the high-risk factors associated with severe disease, ICU admission, and high mortality caused by COVID-19 [12–16]. Therefore, the elderly are accepted as the group with the first priority for vaccination. However, elderly individuals have been reported to develop poor immune responses upon vaccination, even when mRNA vaccines are used [17, 18]. In this study, we are interested in evaluating immune responses (both humoral and cellular immunity) after boosting with either the ChAdOx-1 or BNT162b2 vaccine in older HCWs previously immunized with the 1st series of the CoronaVac vaccine. The levels of NAbs and T-cell responses (i.e., IL-17, IFN-γ, and FasL levels) against the SARS-CoV-2 WT, Alpha, Beta, Delta and Omicron variants were assessed before and after vaccine boosting.

## Methods

### Materials

FITC-labelled anti-CD3, PerCP-anti-CD4, BV785-anti-CD8, PECy7-anti-IFN-γ, BV421-anti-Fas ligand (FasL), and PE-anti-IL-17A monoclonal antibodies (mAbs) were purchased from BioLegend (San Diego, CA, USA). The PepTivator® SARS-CoV-2 Prot_S Complete Pool, SARS-CoV-2 Prot_S B.1.1.7 Mutation Pool, SARS-CoV-2 Prot_S B.1.1.7 WT Reference Pool, SARS-CoV-2 Prot_S B.1.351 Mutation Pool, SARS-CoV-2 Prot_S B.1.351 WT Reference Pool, SARS-CoV-2 Prot_S B.1.617.2 Mutation Pool and SARS-CoV-2 Prot_S B.1.617.2 WT Reference Pool were obtained from Miltenyi Biotec (Bergisch Gladbach, Germany). Brefeldin A and monensin were purchased from Sigma-Aldrich (St. Louis, MO, USA).

### Study design and participants

This prospective single-centre study was performed at Chiang Mai University Hospital, Chiang Mai, Thailand. Forty-six healthy HCWs aged ≥60 years who received 2 doses of the standard CoronaVac regimen in the previous 4–12 weeks were enrolled from 30th July - 1st September 2021. The exclusion criteria were a history of infection with SARS-CoV-2, contact with COVID-19 patients within 2 weeks prior to enrolment, receiving other SARS-CoV-2 vaccines, receiving a live attenuated vaccine in the past 28 days, receiving other inactivated or subunit vaccines in the past 14 days, and a history of allergy to any study vaccine components. Subjects with uncontrolled underlying diseases (e.g., diabetes, cardiovascular disease, pulmonary disease, end-stage renal disease requiring dialysis, or cirrhosis), immunocompromised hosts, and subjects receiving immunosuppressive agents were also excluded. This study was approved by the Ethical Committee of the Faculty of Medicine, Chiang Mai University (IRB approval number: MED-2564-08247), and was filed in the Clinical Trials Registry (Study ID: TCTR20210822002). Before enrolment, written informed consent was obtained from all subjects.

### Study procedures

The subjects were enrolled before the booster vaccination date. All volunteers made their own decision about whether to be boosted with ChAdOx-1 or BNT162b2. The collected general data included demographic data (age, sex, body mass index), smoking status, underlying diseases, medications used, history of vaccinations, COVID-19 exposure risk, dates of prior CoronaVac vaccination and expected dates of booster vaccination. Heparinized blood was taken immediately prior to booster vaccination and at 4 weeks after booster vaccination. The plasma of all participants was tested to determine Nab levels. Ten participants from each age- and sex-matched booster vaccine group were randomly selected and processed for the determination of T-cell responses.

### Assay for determining antibody levels against the SARS-CoV-2 receptor binding domain

Antibodies against the spike receptor binding domain (RBD) of SARS-CoV-2 were quantitatively measured by the SARS-CoV-2 IgG II Quant chemiluminescence immunoassay using the ARCHITECT i System (Abbott Laboratories, Abbot Park, IL, USA). The levels of anti-RBD IgG antibodies were presented in arbitrary units (AU/mL). The obtained AU/mL values were then converted into WHO international standard concentrations (binding antibody units/mL, BAU/mL) following the equation provided by the manufacturer (BAU/mL = 0.142 x AU/mL). Antibody levels greater than or equal to the cut-off value of 7.1 BAU/mL (50 AU/mL) were defined as seropositivity.

### Neutralizing antibody assay

The evaluation of SARS-CoV-2 neutralizing antibodies against WT and VOCs was performed using a cPass SARS-CoV-2 neutralization antibody detection kit (GenScript, NJ, USA). Plasma and positive and negative controls were diluted with sample dilution buffer and preincubated with the horseradish peroxidase (HRP)-labelled RBD protein of the WT, Alpha B.1.1.7, Beta B.1.351, Delta B.1.617.2, or Omicron B.1.1.529 variant at 37 °C for 30 min. The mixtures were then added to capture plates precoated with the human angiotensin-converting enzyme 2 (ACE2) protein, and the plates were incubated at 37 °C for 15 min. The unbound HRP-RBD proteins were removed by washing, and a 3,3′,5,5′-tetramethylbenzidine (TMB) substrate solution was added, followed by the stop solution. The absorbance was measured at 450 nm using a microtiter plate reader. The % inhibition of NAbs was calculated as follows: [1 – (O.D. value of sample/average O.D. value of duplicated negative control)]× 100. The cut-off for SARS-CoV-2 NAb detection was set at 30% inhibition according to the manufacturer’s instructions (GenScript).

### CD4 and CD8 T-cell response assays

Peripheral blood mononuclear cells (PBMCs) were isolated from heparinized blood by Ficoll-Hypque gradient centrifugation. The PBMCs were then stimulated with different peptide pools, including the SARS-CoV-2 Prot_S Complete (WT) Pool; Prot_S B.1.1.7 Mutation Pool, B.1.617.2 Mutation Pool, and Prot_S B.1.617.2 Mutation Pool (including selectively mutated regions of the S proteins of Alpha, Beta, and Delta variants, respectively); and Prot_S B.1.1.7 WT Reference Pool, Prot_S B.1.617.2 WT Reference Pool, and Prot_S B.1.617.2 WT Reference Pool (homologous peptides of the WT sequences of Alpha, Beta, and Delta variants, respectively), according to the manufacturer’s protocol. Briefly, PBMCs were stimulated with the indicated peptide pools and incubated in a 5% CO_2_ incubator at 37 °C for 2 hours. Brefeldin A (1 μg/mL) and monensin (1 μM) were then added, and the cells were continuously incubated for 4 hours. After incubation, the cells were washed, fixed with 4% paraformaldehyde for 15 minutes at room temperature, and then washed 2 times. For Fc receptor blocking and cell permeabilization, 0.1% saponin containing 10% human blood group AB serum was added, and the cells were incubated at 4 °C for 30 minutes. Thereafter, intracellular immunofluorescence staining was performed using PECy7-anti-IFN-γ, BV421-anti-FasL and PE-anti-IL-17A mAbs together with BV785-anti-CD8, PerCP-anti-CD4 and FITC-anti-CD3 mAbs, with incubation at 4 °C for 30 minutes. The stained cells were washed 2 times with 0.01% saponin and fixed with 1% paraformaldehyde. The expression of IL-17A, IFN-γ and FasL in CD4 and CD8 T cells was measured by using a BD FACSCelesta™ flow cytometer (BD Bioscience, San Jose, CA, USA) and analysed with FlowJo software.

### Statistical analysis

The results obtained as numerical data are expressed as the mean ± standard deviation (SD) or the median and interquartile range (IQR). The results recorded as proportions are expressed as frequencies and percentages. The independent-samples t test and Mann–Whitney U test or the McNemar test was used to compare differences between groups based on parametric or nonparametric data, respectively. Fisher’s exact test was used to compare categorical data. The Wilcoxon signed-rank test was used to compare the nonparametric data before and 4 weeks after boosting in each group. Statistical significance was accepted at a *p* value < 0.05. All statistical analyses were performed using GraphPad Prism software version 9.1.2 (GraphPad Software, San Diego, CA, USA).

## Results

### Study participants

In our study, there were 46 volunteers who received 2 doses of the standard regimen of CoronaVac, with 24 and 22 participants in the ChAdOx-1 and BNT162b2 boosting groups, respectively. The mean ages of the ChAdOx-1 and BNT162b2 group participants were 70.1 ± 8.3 years and 74.6 ± 9.4 years, respectively. The demographic data of the two groups were not significantly different (Table 1). The mean timespans between the 2nd dose of CoronaVac and the booster vaccine were 50.0 ± 8.5 days (min-max; 30–56 days) and 60.7 ± 13.6 days (min-max; 35–81 days) for ChAdOx-1 and BNT162b2, respectively.

**Table 1:** Demographic data of the study population (*N* = 46)

Demographic data	ChAdOx-1 (***N*** = 24)	BNT162b2 (***N*** = 22)	***p*** value
Age (years)	70.1 ± 8.3 (min-max, 64–92)	74.6 ± 9.4 (min-max, 60–97)	0.093
BMI (kg/m^2^)	23.9 ± 2.8	23.3 ± 4.3	0.547
Sex (male)	16 (66.7)	10 (45.5)	0.234
Smoking status			1.000
Nonsmoker	22 (91.7)	20 (90.9)	
Ex-smoker	2 (8.3)	2 (9.1)	
Underlying diseases			0.505
Cardiovascular	11 (45.8)	13 (59.1)	
Respiratory	4 (16.7)	0 (0.0)	
Metabolic	1 (4.2)	2 (9.1)	
Neuromuscular	1 (4.2)	2 (9.1)	
Cardiovascular and respiratory	1 (4.2)	1 (4.5)	
Cardiovascular and metabolic	2 (8.3)	1 (4.5)	
Respiratory and metabolic	1 (4.2)	0 (0.0)	
Respiratory and gastrointestinal	1 (4.2)	0 (0.0)	
None	2 (8.3)	3 (13.6)	

Data are presented as the mean ± SD or N (%)

### Anti-SARS-CoV-2 receptor binding domain antibody responses

**Fig. 1:**
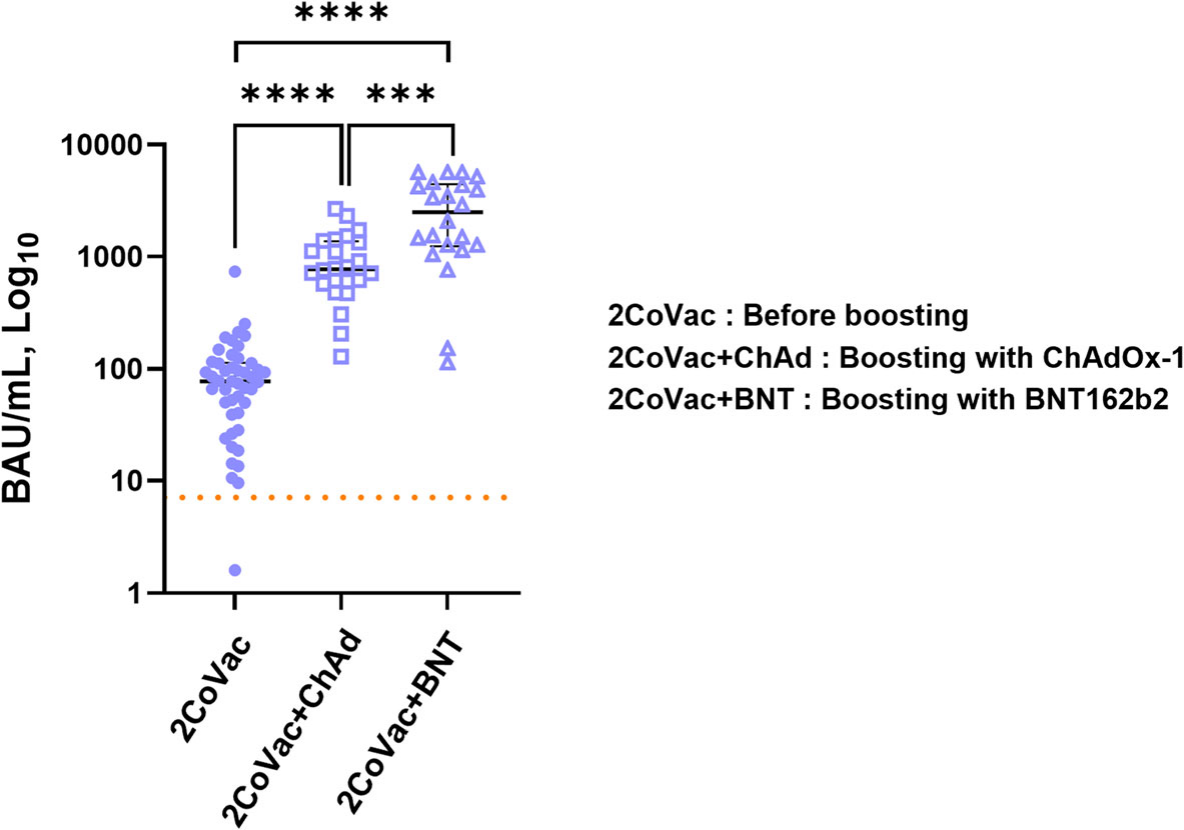
Quantification of anti-SARS-CoV-2 RBD antibodies after booster vaccination. Blood samples were collected after the 2-dose CoronaVac regimen (2CoVac) (*N* = 46) as a baseline before boosting and 4 weeks after boosting with ChAdOx1 (2CoVac + ChAd) (*N* = 24) or BNT162b2 (2CoVac + BNT) (*N* = 22). The scatter plots with the median and interquartile range (IQR) of anti-SARS-CoV-2 RBD IgG levels are shown. The Mann–Whitney U test was used for comparison, *** *p* < 0.001, **** *p* < 0.0001

The levels of anti-SARS-CoV-2 RBD antibodies before and after vaccine boosting were examined. Prior to receiving the booster dose, 45 out of 46 (97.8%) CoronaVac prime participants were seropositive for anti-RBD IgG antibodies, with a median concentration of 77.7 BAU/mL (IQR 42.9–12.3 BAU/mL) (Fig. 1). The antibody levels were dramatically increased after boosting with either ChAdOx-1 or BNT162b2. The antibodies were present in all subjects after boosting (Fig. 1). The median levels of anti-RBD antibodies in the ChAdOx-1 and BNT162b2 groups were 771.0 BAU/mL (IQR 593.8–1344.8) and 2493.0 BAU/mL (IQR 1272.3–4328.5), respectively. The anti-RBD antibody levels in the BNT162b2 group were significantly higher than those in the ChAdOx-1 group (*p* < 0.001) (Fig. 1).

### Neutralizing antibody responses

**Fig. 2:**
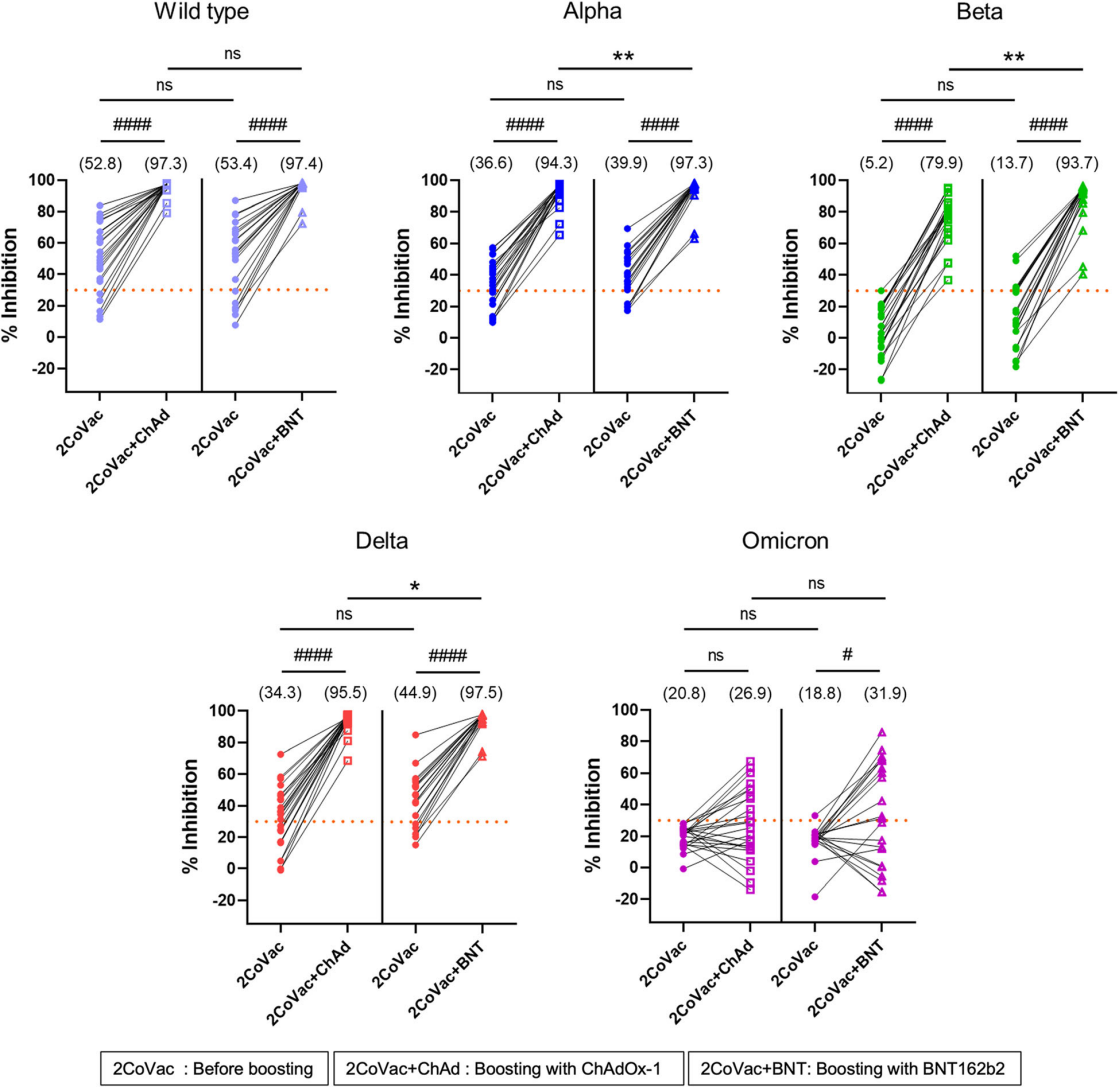
Neutralizing antibodies against the wild-type, Alpha, Beta, Delta, and Omicron variants. Plasma obtained from the study population before (2CoVac) and 4 weeks after boosting with ChAdOx-1(2CoVac + ChAd) (*N* = 24) or BNT162b2 (2CoVac + BNT) (*N* = 22) was used to determine the percent inhibition of neutralizing antibodies against the wild-type, Alpha, Beta, Delta, and Omicron variants. The horizontal lines indicate the 30% inhibition threshold of neutralizing antibodies. Dot plots represent each individual, lines connect data from the same individual on the NAbs against the indicated variants, and medians with interquartile ranges are shown. The numbers in parentheses indicate the median values. The Mann–Whitney U test was used to compare the different samples; * *p* < 0.05, ** *p* < 0.01, ns = not statistically significant. The Wilcoxon signed-rank test was used for comparisons of the paired samples; ^#^ *p* < 0.05, ^####^ *p* < 0.0001

Before boosting, the median % inhibition of NAbs against the WT, Alpha, Beta, Delta, or Omicron variant in the ChAdOx-1 versus BNT162b2 groups was 52.8% vs. 53.4, 36.6% vs. 39.9, 5.2% vs. 13.7, 34.3% vs. 44.9, and 20.8% vs. 18.8%, respectively (Fig. 2 and Table 2). The NAb levels against each variant were not different between the ChAdOx-1 and BNT162b2 groups (Fig. 2). The numbers of subjects who showed a % inhibition of NAbs above the 30% threshold for the WT, Alpha, Beta, Delta, and Omicron variants were 35 (76.1%), 33 (71.7%), 7 (15.2%), 30 (65.2%), and 1 (2.2%), respectively (Table 3).

**Table 2:** Neutralizing antibodies against the SARS-CoV-2 wild type, Alpha, Beta, Delta, and Omicron variants

	ChAdOx-1 group (***N*** = 24)			BNT162b2 group (***N*** = 22)		
	Before boosting	4 weeks after boosting	***p*** value	Before boosting	4 weeks after boosting	***p*** value
**Wild type**	52.8 (36.9–67.2)	97.3 (96.9–97.5)	< 0.0001	53.4 (31.0–66.3)	97.4 (96.9–97.6)	< 0.0001
**Alpha (B.1.1.7)**	36.6 (23.4–44.6)	94.3 (92.3–96.6)	< 0.0001	39.9 (30.8–51.0)	97.3 (94.8–97.9)	< 0.0001
**Beta (B.1.351)**	5.2 (−5.4–18.7)	79.9 (68.9–84.7)	< 0.0001	13.7 (3.6–30.5)	93.7 (88.5–95.4)	< 0.0001
**Delta (B.1.617.2)**	34.3 (17.1–44.7)	95.5 (93.8–96.9)	< 0.0001	44.9 (29.9–53.3)	97.5 (95.0–97.9)	< 0.0001
**Omicron (B.1.1.529)**	20.8 (14.9–24.4)	26.9 (12.9–46.4)	0.0828	18.8 (16.9–20.5)	31.9 (3.6–66.7)	0.0275

Data are presented as the median (interquartile range, IQR)

Neutralizing antibodies (% inhibition) against the SARS-CoV-2 wild-type, Alpha, Beta, Delta, and Omicron variants before and 4 weeks after boosting with ChAdOx-1 and BNT162b2 in elderly health care workers

**Table 3:** Numbers of subjects who had neutralizing antibody levels above the 30% inhibition threshold

	All (***N*** = 46)	ChAdOx-1 group (***N*** = 24)			BNT162b2 group (***N*** = 22)		
	Before boosting	Before boosting	4 weeks after boosting	***p*** value	Before boosting	4 weeks after boosting	***p*** value
**Wild type**	35 (76.1)	19 (79.2)	24 (100.0)	0.062	16 (72.7)	22 (100.0)	0.031
**Alpha (B.1.1.7)**	33 (71.7)	16 (66.7)	24 (100.0)	0.008	17 (77.3)	22 (100.0)	0.062
**Beta (B.1.351)**	7 (15.2)	0 (0)	24 (100.0)	< 0.001	7 (31.8)	22 (100.0)	< 0.001
**Delta (B.1.617.2)**	30 (65.2)	14 (58.3)	24 (100.0)	0.002	16 (72.7)	22 (100.0)	0.031
**Omicron (B.1.1.529)**	1 (2.2)	0 (0)	10 (41.7)	0.002	1 (4.5)	12 (54.5)	0.001

Data are presented as N (%)

The numbers of elderly health care workers who had levels of neutralizing antibodies against the SARS-CoV-2 wild-type, Alpha, Beta, Delta, and Omicron variants above the 30% inhibition threshold before and 4 weeks after boosting with the ChAdOx-1 and BNT162b2 vaccines

Four weeks after boosting with ChAdOx-1 or BNT162b2, the median % inhibition of NAbs against the WT, Alpha, and Delta variants increased to more than 94% (range 94.3–97.5%) (Table 2 and Fig. 2). The increase in NAbs against the Beta variant, however, was less than those against the other variants. The median % inhibition of NAbs against the Beta variant was 79.9 and 93.7% for ChAdOx-1 and BNT162b2, respectively (Table 2 and Fig. 2). After boosting with either ChAdOx-1 or BNT162b2, the NAbs against the WT, Alpha, Beta, and Delta variants were highly significantly increased from the baseline (*p* < 0.0001) (Fig. 2 and Table 2). NAb levels against the Alpha, Beta, and Delta variants, but not the WT, were significantly higher in the BNT162b2 group than in the ChAdOx-1 group (Fig. 2). After boosting with the ChAdOx-1 and BNT162b2 vaccines, all individuals showed a % inhibition of NAbs against the WT, Alpha, Beta, and Delta variants above the 30% threshold (Table 3).

Before boosting, the levels of NAbs against the Omicron variant were lower than those against other variants (Fig. 2). After vaccine boosting, the median % inhibition of NAbs against Omicron was 26.9% for ChAdOx-1 and 31.9% for BNT162b2, which were also lower than the values for the other variants (Fig. 2 and Table 2). The percentages of subjects who showed a % inhibition of NAbs above the 30% threshold for the Omicron variant were 41.7 and 54.5% for ChAdOx-1 and BNT162b2, respectively (Table 3). The levels of NAbs against Omicron were not significantly different before and after boosting with either ChAdOx-1 or BNT162b2 or between the groups boosted with ChAdOx-1 and BNT162b2 (Table 2 and Fig. 2).

### CD4 and CD8 T-cell responses

Overlapping peptide pools derived from the S proteins of the WT, Alpha, Beta, and Delta variants were used to stimulate PBMCs collected before and after boosting with ChAdOx-1 or BNT162b2. After stimulation, CD4 and CD8 T cells were examined to evaluate the expression of IL-17A, IFN-γ, and FasL using flow cytometry. The gating strategy is shown in Additional file 1 (Figure S1).

**Fig. 3:**
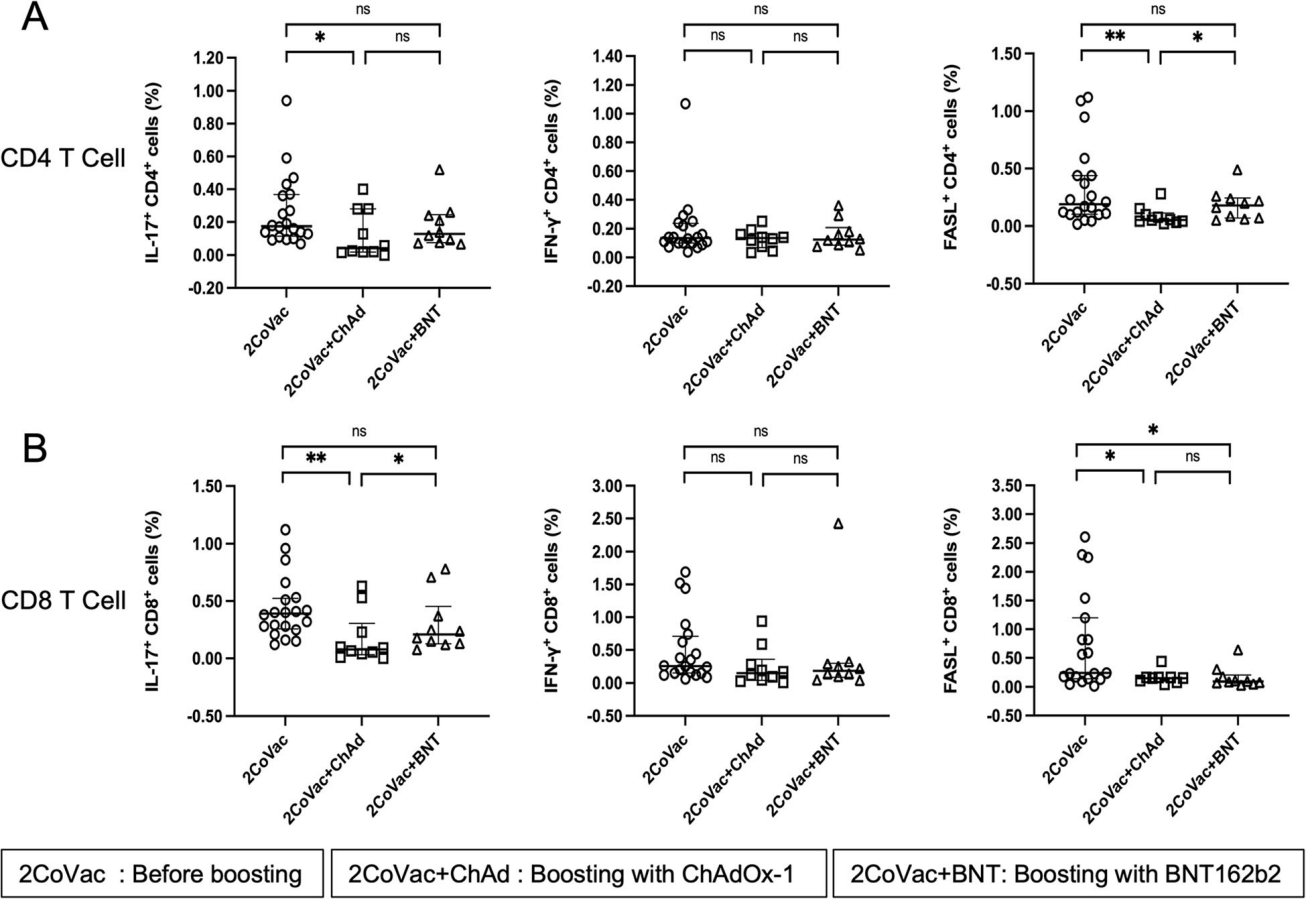
T-cell responses to pooled peptides of the SARS-CoV-2 wild-type spike protein. PBMCs were stimulated with pooled peptides of the wild-type spike protein (all functional domains). The frequency of CD4 T cells (**A**) or CD8 T cells (**B**) expressing IL-17A, IFN-γ or FasL before (2CoVac) (*N* = 20) and 4 weeks after boosting with ChAdOx-1 (2CoVac + ChAd) (*N* = 10) and BNT162b2 (2CoVac + BNT) (*N* = 10) was determined using intracellular immunofluorescence staining and flow cytometry. Individual data points are shown. Lines represent the median with the interquartile range. The Mann–Whitney U test was used for comparisons. ns indicates no significant difference (*p* > 0.05), * *p* ≤ 0.05 and ** *p* ≤ 0.01

Upon activation with WT S peptides (including all functional domains), IL-17A production in CD4 T cells was not different before and after boosting with BNT162b2 but was reduced in the postboosting ChAdOx-1 group (Fig. 3A). Similar observations were made in CD8 T cells (Fig. 3B). When the postboosting results were compared between the ChAdOx-1 and BNT162b2 groups, a trend towards higher IL-17A production in both CD4 and certain CD8 T cells was observed in the BNT162b2 group versus the ChAdOx-1 group (Fig. 3A and B). Notably, in CD4 T cells, the IL-17A level postboosting was not significantly different between the BNT162b2 and ChAdOx-1 groups (Fig. 3A). However, IFN-γ production by CD4 and CD8 T cells was not significantly different before and after boosting under all vaccination regimens (Fig. 3A and B). Interestingly, FasL expression in CD4 T cells was significantly higher in the BNT162b2-boosted group than in the ChAdOx-1-boosted group (Fig. 2A). This phenomenon was not observed in CD8 T cells (Fig. 3B). However, FasL expression was higher before boosting than after vaccine boosting (Fig. 3). Notably, FasL expression in CD4 T cells was not significantly different before and after boosting with BNT162b2 (Fig. 3A). The results indicated that, upon stimulation with WT S peptides, a trend towards higher IL-17A and FasL production were observed in the BNT162b2 group than in the ChAdOx-1 group.

**Fig. 4:**
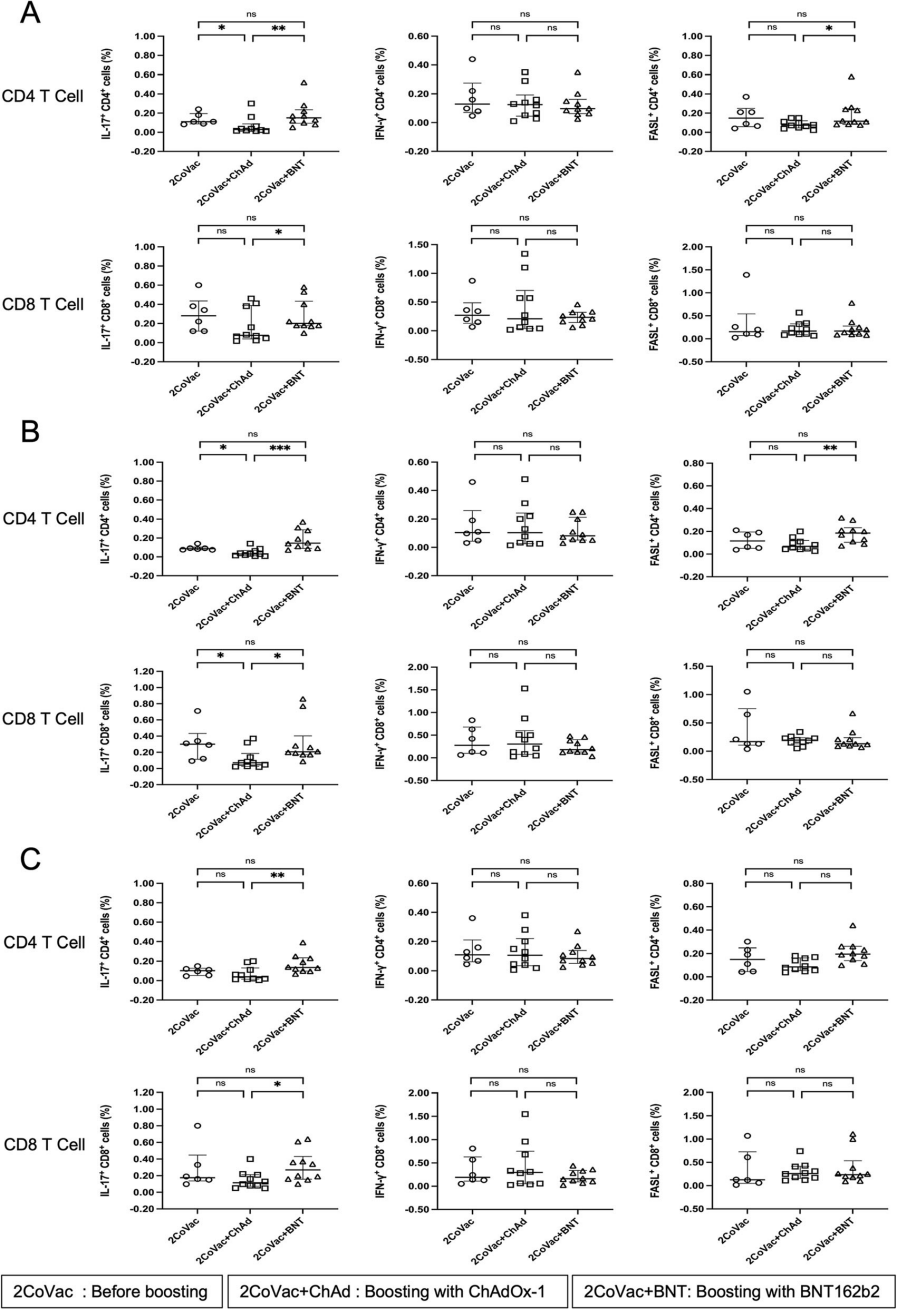
T-cell responses to pooled peptides of SARS-CoV-2 variant spike proteins. PBMCs were stimulated with pooled peptides of SARS-CoV-2 variant spike proteins, including the following pools: **A** B.1.1.7 mutation; Alpha variant, **B** B.1.351 mutation; Beta variant, or **C** B.1.617.2 mutation; Delta variant. The frequency of CD4 or CD8 T cells expressing IL-17A, IFN-γ or FasL before (2CoVac) (*N* = 6) and 4 weeks after boosting with ChAdOx-1 (2CoVac + ChAd) (*N* = 10) and BNT162b2 (2CoVac + BNT) (*N* = 10) was determined using intracellular immunofluorescence staining and flow cytometry. Individual data points are shown. Lines represent the median with the interquartile range. The Mann–Whitney U test was used for comparison. ns indicates no significant difference (*p* > 0.05), * *p* ≤ 0.05, ** *p* ≤ 0.01, and*** *P* < 0.0005

We also investigated the T-cell response to the S peptides of the Alpha, Beta, and Delta variants. As shown in Fig. 4, upon activation, IL-17A, IFN-γ, and FasL production in both CD4 and CD8 T cells showed the same pattern observed upon WT S peptide stimulation. Stimulation with the S peptides of all tested VOCs produced similar IL-17A, IFN-γ, and FasL production profiles (Fig. 4A-C).

Our experiments included stimulation with the WT homologues of each VOC peptide. As shown in the Additional files (Figure S2 and S3), stimulation by mutant VOC peptides and their WT homologues resulted in no significant differences in the expression of IL-17A, IFN-γ, and FasL in CD4 and CD8 T cells.

## Discussion

Ageing is a risk factor associated with not only severe COVID-19 disease but also a poor immune response to vaccination. Immunosenescence due to age-related changes in the counts and functions of T cells is the major underlying mechanism [19]. In elderly individuals, immunization with inactivated vaccine results in weak humoral and cellular immune responses [20–23]. Therefore, we put forth the question of whether an inactivated vaccine for SARS-CoV-2 could induce adequate immune responses, particularly for VOCs, in elderly individuals and investigated the beneficial effects of boosting with virus-vectored and mRNA vaccines.

In the present study, we determined the levels of antibodies against the RBD of SARS-CoV-2 before and after vaccine boosting in elderly individuals previously vaccinated with 2 doses of the CoronaVac inactivated vaccine. After boosting with either ChAdOx-1 or BNT16b2, anti-RBD antibody levels were dramatically increased; however, the antibody level was significantly higher in the BNT16b2 group than in the ChAdOx-1 group. BNT16b2 induced higher humoral immunity than ChAdOx-1 [24]. Nevertheless, the occurrence of breakthrough infections with SARS-CoV-2 in HCWs was correlated with NAb levels [25].

In addition to anti-RBD antibodies, we measured the levels of NAbs against the SARS-CoV-2 virus, including the ancestral type and the Alpha, Beta, Delta, and Omicron VOCs. Nab levels are a more precise predictor of adequate immunity against SARS-CoV-2 infection [26]. Unfortunately, the protective level of antibody for prevention of COVID-19 is currently unknown. We used 30% inhibition as the cut-off level for defining the detection of NAbs, as suggested by the manufacturer of the kit used for antibody detection. Our study demonstrated that 1–3 months after completing the 2-dose CoronaVac immunization regimen, the percentages of subjects showing a % inhibition of NAbs above the 30% threshold were 76.1, 71.7, 15.2%, 65.2, and 2.2% for the WT, Alpha, Beta, Delta, and Omicron variants, respectively. If we were to assume that a % inhibition over 30% represents a protective level, 34.8 and 97.8% of CoronaVac-vaccinated individuals would be at high risk for Delta and Omicron infections, respectively. Our study is complementary to previous reports of studies in young adults [27, 28]. Although high effectiveness in the prevention of hospitalization and death may be maintained among young HCWs, this might not be the case in older populations. Importantly, any SARS-CoV-2 infections that arise among HCWs will have significant impacts on the patients’ safety and the health care system, regardless of disease severity. Therefore, booster vaccination with other vaccine platforms to increase NAb level to prevent infections with SARS-CoV-2 variants among HCWs, particularly in the elderly, would be reasonable. In Thailand, the approved viral-vectored and mRNA vaccines available during our study period were ChAdOx-1 and BNT162b2, respectively. Comparison of the effectiveness of these 2 vaccines following the primary vaccination series showed that the BNT162b2 vaccine was superior to the ChAdOx-1 vaccine [29–34]. However, the data available for comparing the immunogenicity achieved using ChAdOx-1 or BNT162b2 as the booster vaccine in elderly individuals following priming with the CoronaVac inactivated vaccine are limited.

In the present study, boosting elderly individuals with ChAdOx1 or BNT162b2 following a standard 2-dose CoronaVac regimen resulted in a significant increase in NAb levels against the WT, Alpha, Beta, and Delta variants. However, BNT162b2 induced significantly higher NAb levels than ChAdOx-1 for Alpha, Beta, and Delta variants. Before boosting, the levels of NAbs against SARS-CoV-2 variants were not significantly different between these two groups. Our results were in accords with another similar study focussing on a younger age group (18–60 years) [24]. Although ChAdOx-1 boosting induced lower NAb levels than BNT162b2 boosting, the former vaccine still induced a high-level antibody response. In this study, all individuals developed NAbs against the WT, Alpha, Beta, and Delta variants after boosting with ChAdOx-1. Thus, the ChAdOx-1 vaccine would be beneficial for use as a boosting vaccine.

In November 2021, a new SARS-CoV-2 variant, Omicron, was reported by the WHO [10]. The Omicron variant shows several mutated sites within the S protein [35], including 15 mutations in the RBD region and 8 mutations are in the N-terminal domain (NTD). Importantly, these mutation sites are the immunodominant targets of NAbs elicited by SARS-CoV-2 infection or COVID-19 vaccines [36, 37]. In accord with expectations, after immunization with the primary 2-dose CoronaVac regime, NAbs against the Omicron variant were detected in only 2 out of 46 (2.2%) vaccinated individuals. These results indicated that the inactivated vaccine may not be sufficient to protect against this VOC. Nevertheless, boosting with either ChAdOx1 or BNT162b2 following 2 doses of CoronaVac induced lower levels of NAbs against the Omicron variant than against the other VOCs. Approximately 50% of vaccine-boosted subjects showed NAb levels against the Omicron variant below the 30% threshold. Our results were similar to a previous study showing that boosting with BNT162b2 in vaccinees primed with ChAdOx-1/ChAdOx-1 or ChAdOx-1/BNT162b2 or the BNT162b2/BNT162b2 regimen induced low levels of NAbs against the Omicron variant [38]. This phenomenon might explain the new wave of global outbreaks caused by this variant despite the large numbers of people who are fully vaccinated.

The effectiveness of vaccines primarily depends on the induction of high levels of neutralizing antibodies; however, T cells also play critical roles in virus elimination. In this study, we also evaluated the CD4 and CD8 T-cell responses of our elderly participants. We compared the expression levels of the cytokines IL-17A and IFN-γ and FasL in both CD4 and CD8 T cells upon stimulation with the S peptides of the WT virus and VOCs. IL-17A is a cytokine produced by a wide range of immune cell types, including CD4 and CD8 T cells [39, 40]. This cytokine plays a crucial role in enhancing effective antiviral immune responses [41, 42]. IL-17 hinders viral infection via several mechanisms, including enhancing the Th1 immune response, promoting cytotoxic T cell functions and inducing protective inflammatory responses [41]. FasL (CD95L or CD178) is a type-II transmembrane protein expressed on cytotoxic cells. Cytotoxic T cells utilize FasL to kill target cells displaying the relevant MHC-bound peptide [43, 44]. We speculated that the induction of IL-17 and FasL in T cells after vaccination might play an important role in immunity to SARS-CoV-2 infection, particularly for VOCs. IFN-γ is a pleiotropic cytokine that plays a role in protection against viral infection by regulating effector cells in both innate and adaptive immunity [45–47]. Several IFN-γ-orchestrated mechanisms contributing to the host antiviral response have been reported [45]. Therefore, the effects of vaccination on the induction of IL-17A, IFN-γ, and FasL in CD4 and CD8 T cells were determined in our experiments.

Interestingly, stimulation with S peptides derived from the Alpha, Beta and Delta variants resulted in significantly higher expression levels of IL-17A in CD8 T cells and FasL in CD4 T cells in the BNT162b2-boosted group than in the ChAdOx-1-boosted group. Both BNT162b2 and ChAdOx-1 are genetic vaccines driving SARS-CoV-2 S protein biosynthesis in body cells. However, substantial differences between mRNA and adenovirus-vector vaccines that can affect the conformation of the S protein produced and its presentation to the immune system have been reported [48, 49]. These factors might affect the ability of vaccines to stimulate immune responses. The reduction in IL-17A in the postboosting ChAdOx-1 group compared with the level before boosting may have resulted from the time that passed between receiving the second dose of CoronaVac and blood collection after vaccine boosting. Nevertheless, the exact molecular mechanisms need to be elucidated. To the best of our knowledge, these findings have never been reported previously. Although CD8 T cells are traditionally considered to be cytotoxic T cells, it has become clear that CD4 T cells (usually Th1 cells) can also show direct cytotoxicity [50–52]. The FasL-Fas interaction of CD4 T cells and target cells has been suggested to be involved in CD4 T-cell-mediated cytotoxicity [50]. Cytotoxic CD4 T cells are induced during viral infection and vaccination [53–55]. The induction of FasL in CD4 T cells upon BNT162b2 boosting might be involved in protective immunity against COVID-19.

IFN-γ production by the CD4 and CD8 T cells of our elderly participants did not significantly differ under any of the tested vaccination regimens. Our results are not in agreement with previous reports [24, 56]. This discrepancy was not surprising and could be due to the methods and subjects of the different studies. The interferon-γ release assay (IGRA) was employed in previous studies, [24, 56] while intracellular IFN-γ expression in CD4 and CD8 T cells [57] was evaluated in the present study. Through the intracellular cytokine assay, the numbers of IFN-γ-producing CD4 and CD8 T cells could be directly monitored upon stimulation with S peptide pools. The antigens used in the present and previous reports were also different, and the results obtained cannot be compared. In addition, the study subjects were different, as healthy adults aged 18–60 years were examined the previous studies [24, 56], while elderly individuals were examined in this study. Immune dysfunction in the elderly caused by reduced T-cell function has been demonstrated [19].

In conclusion, our study focusing on the immunogenicity of booster vaccination against SARS-CoV-2 VOCs in elderly individuals suggested that booster immunization with either ChAdOx-1 or BNT162b2 following inactivated virus vaccination resulted in a significant increase in the NAb response. The induction of NAbs against the Omicron variant was, however, weaker than that against other VOCs. The next question to be studied is how long the immune responses induced by the booster vaccines will last. There are some limitations of our study. First, the number of subjects was low. The main reasons for this were that the number of elderly HCWs actively working in our institute was limited, and the booster regimen was initially allowed only in HCWs, not in the general population. Second, this study was not a double-blinded randomized control trial. The subjects could select their own studied booster vaccines and there was no non-vaccine booster control in our study. Therefore, the selection bias would be possible and data on the immunologic differences between boosting and non-boosting volunteers could not be obtained, respectively. Third, although the levels of NAbs against the SARS-CoV-2 WT and variant strains were determined in this study, neither pseudovirus nor live-virus neutralization assays were performed.

## Supplementary Information

**Additional file 1: Figure S1.** Flow cytometry and gating strategy. PBMCs were stimulated with the SARS-CoV-2 peptide pool of spike proteins, and IL-17A, IFN-γ and FasL expression levels were determined by flow cytometry. The gating strategies are shown. **(A)** The size (FSC-A) and granularity (SSC-A) of PBMCs were plotted and gated as indicated. **(B)** The gated cells were represented in an SSC-H vs. SSC-A dot plot to eliminate doublets. **(C)** CD3 T cells were gated by plotting CD3 staining vs. SSC-A. **(D)** CD4 T cells and CD8 T cells were gated from CD3 T cells by plotting CD8 vs. CD4 staining. The CD4 T cells **(E)** and CD8 T cells **(F)** were plotted against CD4 or CD8 staining and IL-17A, IFN-γ or FasL expression. IFN-γ is shown as an example in this figure. The percentages of IL-17A-, IFN-γ- or FasL-producing cells were investigated. **Figure S2.** T-cell responses to wild-type spike peptide homology with the mutated regions in spike variants B.1.1.7, B.1.351 and B.1.617.2. PBMCs were stimulated with a pool of wild-type spike peptides showing homology to mutated regions in the spike proteins of **(A)** B.1.1.7 mutation; Alpha variants, **(B)** B.1.351 mutation; Beta variants, or **(C)** B.1.617.2 mutation; Delta variants. Immunofluorescence staining and flow cytometry were used to determine the frequency of CD4 or CD8 T cells expressing IL-17A, IFN-γ or FasL. Individual data points before (2CoVac) (*N* = 6) and 4 weeks after boosting with ChAdOx-1 (2CoVac + ChAd) (*N* = 10) or BNT162b2 (2CoVac + BNT) (*N* = 10) are shown. Lines represent the median with the interquartile range. The Mann–Whitney U test was used for comparison. ns indicates no significant difference (*p* > 0.05), * *p* ≤ 0.05, ** *p* ≤ 0.01, and*** *P* < 0.0005. **Figure S3.** Comparison of CD4 and CD8 T-cell responses to wild-type spike peptides homology with the mutated regions of spike variants B.1.1.7, B.1.351 and B.1.617.2. PBMCs were stimulated with spike peptide pools consisting of B.1.1.7 (Alpha), B.1.351 (Beta), or B.1.617.2 (Delta) mutants and their homologous WT peptides. Immunofluorescence staining and flow cytometry were used to determine the frequency of CD4 or CD8 T cells expressing **(A)** IL-17A, **(B)** IFN-γ, and **(C)** FasL. The assays were evaluated before (2CoVac) (*N* = 6) and after boosting with ChAdOx-1 (2CoVac + ChAd) (*N* = 10) or BNT162b2 (2CoVac + BNT) (*N* = 10). The Wilcoxon matched pairs signed-ranks test was used for comparison. ns indicates no significant difference (*p* > 0.05), and * represents *p* ≤ 0.05.

## Data Availability

Data generated or analysed during this study are included in this published article or its Supplementary Information files and are available from the corresponding author upon reasonable request.

## Abbreviations

BV: Brilliant Violet
Cy: Cyanine
FasL: Fas Ligand
FITC: Fluorescein isothiocyanate
IL: Interleukin
IFN-γ: Interferon-γ
PE: Phycoerythrin
PerCP: Peridinin-Chlorophyll-Protein
