# Supplementary material for: Neutralizing antibody and T cell responses against SARS-CoV-2 variants of concern following ChAdOx-1 or BNT162b2 boosting in the elderly previously immunized with CoronaVac vaccine
Source: Immun Ageing. 2022 May 24;19:24. doi: 10.1186/s12979-022-00279-8 (PMC9126751; doi:10.1186/s12979-022-00279-8)
Supplement: Supplementary file 1 — Additional file 1: Figure S1. Flow cytometry and gating strategy. PBMCs were stimulated with the SARS-CoV-2 peptide pool of spike proteins, and IL-17A, IFN-γ and FasL expression levels were determined by flow cytometry. The gating strategies are shown. (A) The size (FSC-A) and granularity (SSC-A) of PBMCs were plotted and gated as indicated. (B) The gated cells were represented in an SSC-H vs. SSC-A dot plot to eliminate doublets. (C) CD3 T cells were gated by plotting CD3 staining vs. SSC-A. (D) CD4 T cells and CD8 T cells were gated from CD3 T cells by plotting CD8 vs. CD4 staining. The CD4 T cells (E) and CD8 T cells (F) were plotted against CD4 or CD8 staining and IL-17A, IFN-γ or FasL expression. IFN-γ is shown as an example in this figure. The percentages of IL-17A-, IFN-γ- or FasL-producing cells were investigated. Figure S2. T-cell responses to wild-type spike peptide homology with the mutated regions in spike variants B.1.1.7, B.1.351 and B.1.617.2. PBMCs were stimulated with a pool of wild-type spike peptides showing homology to mutated regions in the spike proteins of (A) B.1.1.7 mutation; Alpha variants, (B) B.1.351 mutation; Beta variants, or (C) B.1.617.2 mutation; Delta variants. Immunofluorescence staining and flow cytometry were used to determine the frequency of CD4 or CD8 T cells expressing IL-17A, IFN-γ or FasL. Individual data points before (2CoVac) (N = 6) and 4 weeks after boosting with ChAdOx-1 (2CoVac + ChAd) (N = 10) or BNT162b2 (2CoVac + BNT) (N = 10) are shown. Lines represent the median with the interquartile range. The Mann–Whitney U test was used for comparison. ns indicates no significant difference (p > 0.05), * p ≤ 0.05, ** p ≤ 0.01, and*** P < 0.0005. Figure S3. Comparison of CD4 and CD8 T-cell responses to wild-type spike peptides homology with the mutated regions of spike variants B.1.1.7, B.1.351 and B.1.617.2. PBMCs were stimulated with spike peptide pools consisting of B.1.1.7 (Alpha), B.1.351 (Beta), or B.1.617.2 (Delta) muta [file 12979_2022_279_MOESM1_ESM.docx]

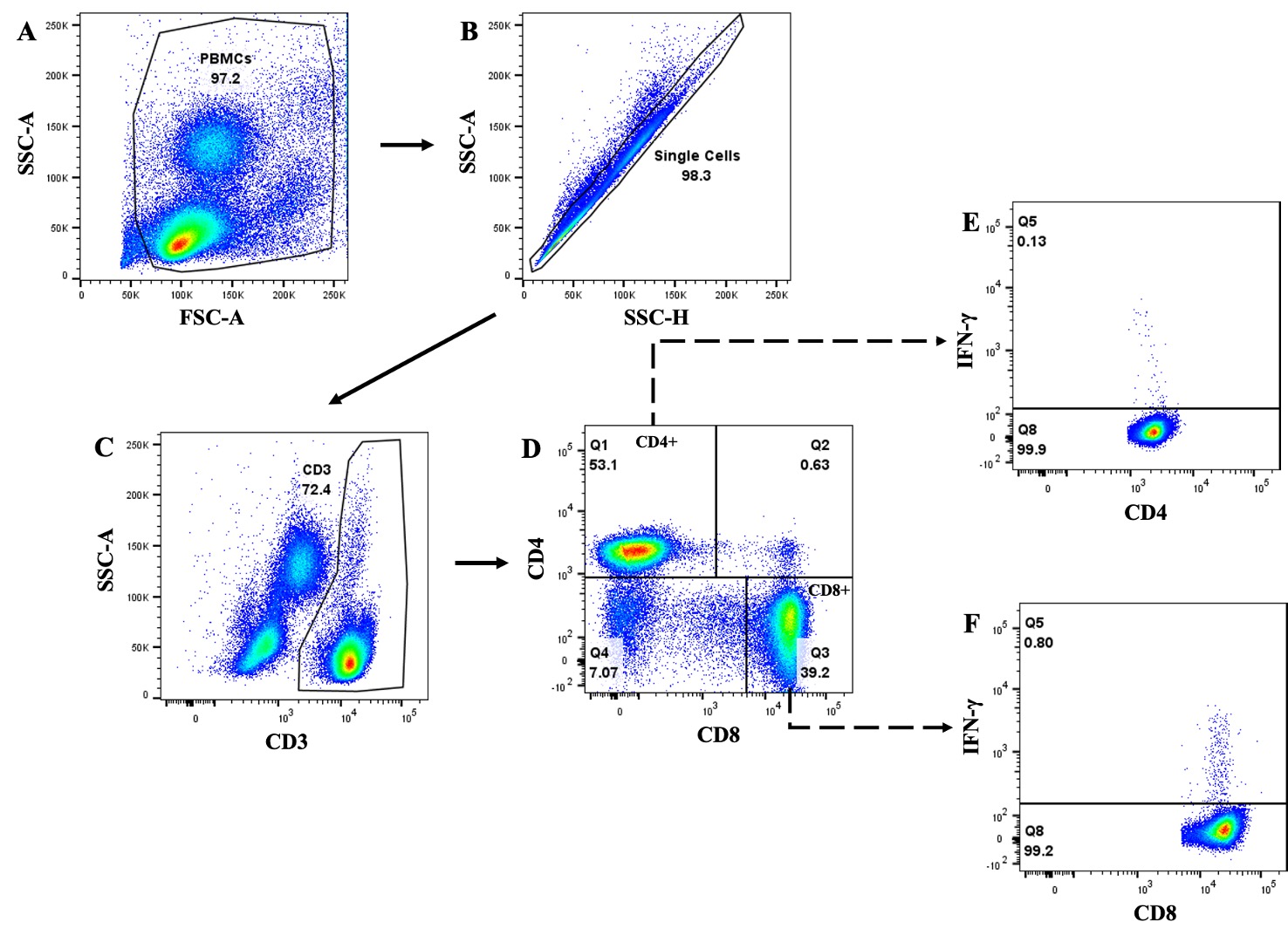


**Figure S1. Flow cytometry and gating strategy.** PBMCs were stimulated with the SARS-CoV-2 peptide pool of spike proteins, and IL-17A, IFN-γ and FasL expression levels were determined by flow cytometry. The gating strategies are shown. **(A)** The size (FSC-A) and granularity (SSC-A) of PBMCs were plotted and gated as indicated. **(B)** The gated cells were represented in an SSC-H vs. SSC-A dot plot to eliminate doublets. **(C)** CD3 T cells were gated by plotting CD3 staining vs. SSC-A. **(D)** CD4 T cells and CD8 T cells were gated from CD3 T cells by plotting CD8 vs. CD4 staining. The CD4 T cells **(E)** and CD8 T cells **(F)** were plotted against CD4 or CD8 staining and IL-17A, IFN-γ or FasL expression. IFN-γ is shown as an example in this figure. The percentages of IL-17A-, IFN-γ- or FasL-producing cells were investigated.


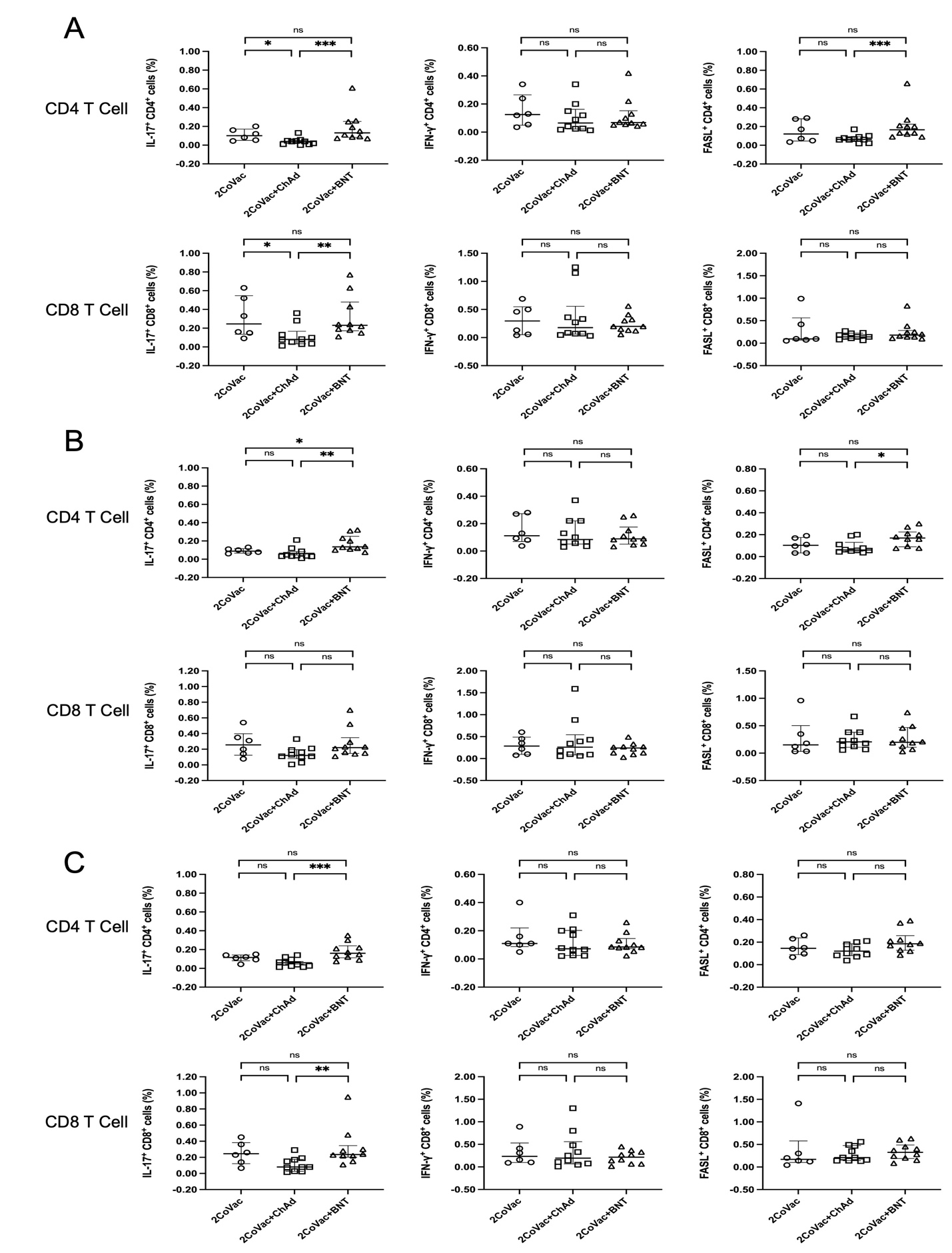


**Figure S2. T-cell responses to wild-type spike peptide homology with the mutated regions in spike variants B.1.1.7, B.1.351 and B.1.617.2.** PBMCs were stimulated with a pool of wild-type spike peptides showing homology to mutated regions in the spike proteins of **(A)** B.1.1.7 mutation; Alpha variants, **(B)** B.1.351 mutation; Beta variants, or **(C)** B.1.617.2 mutation; Delta variants. Immunofluorescence staining and flow cytometry were used to determine the frequency of CD4 or CD8 T cells expressing IL-17A, IFN-γ or FasL. Individual data points before (2CoVac) (N=6) and 4 weeks after boosting with ChAdOx-1 (2CoVac+ChAd) (N=10) or BNT162b2 (2CoVac+BNT) (N=10) are shown. Lines represent the median with the interquartile range. The Mann–Whitney U test was used for comparison. ns indicates no significant difference (p > 0.05), * p ≤ 0.05, ** p ≤ 0.01, and*** P<0.0005.


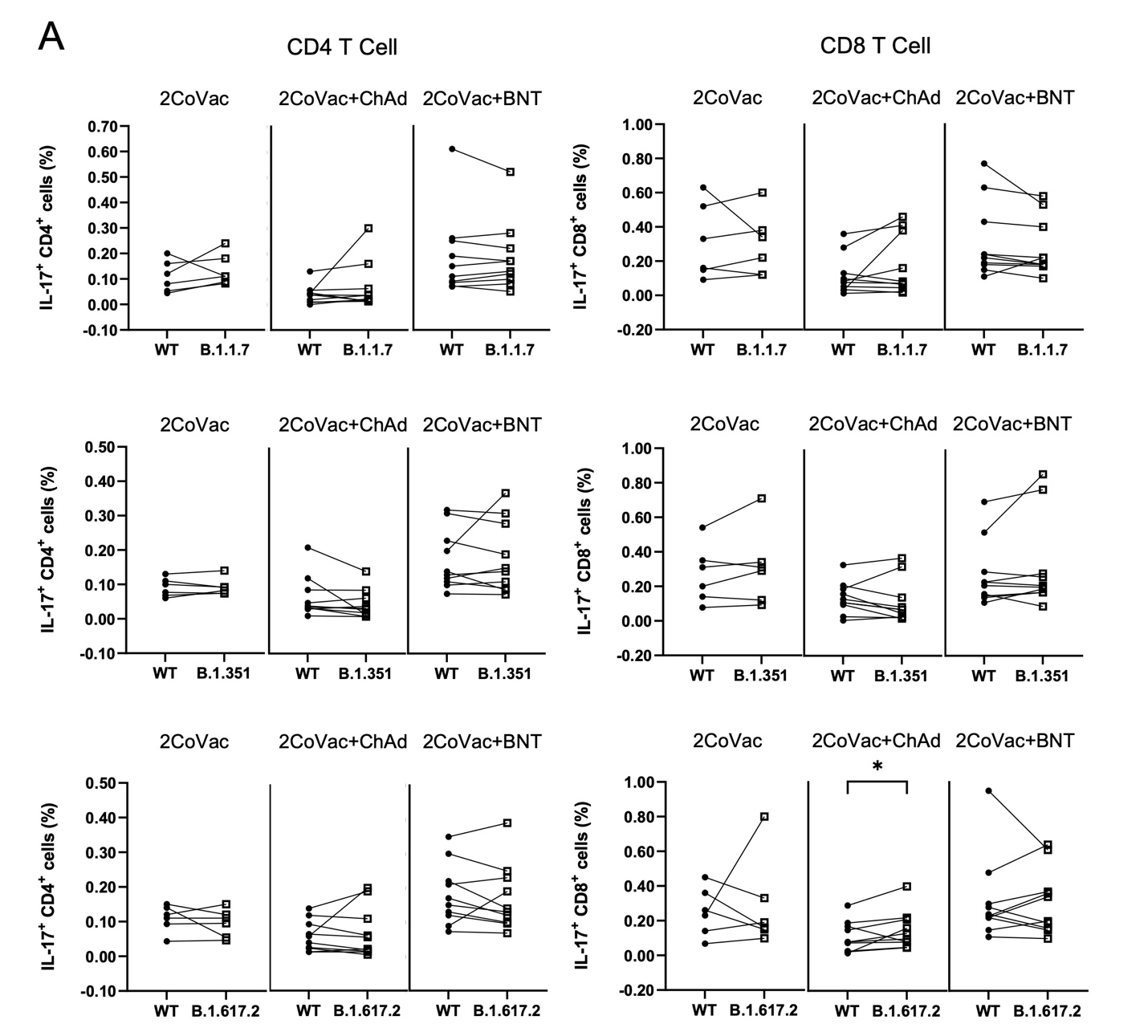


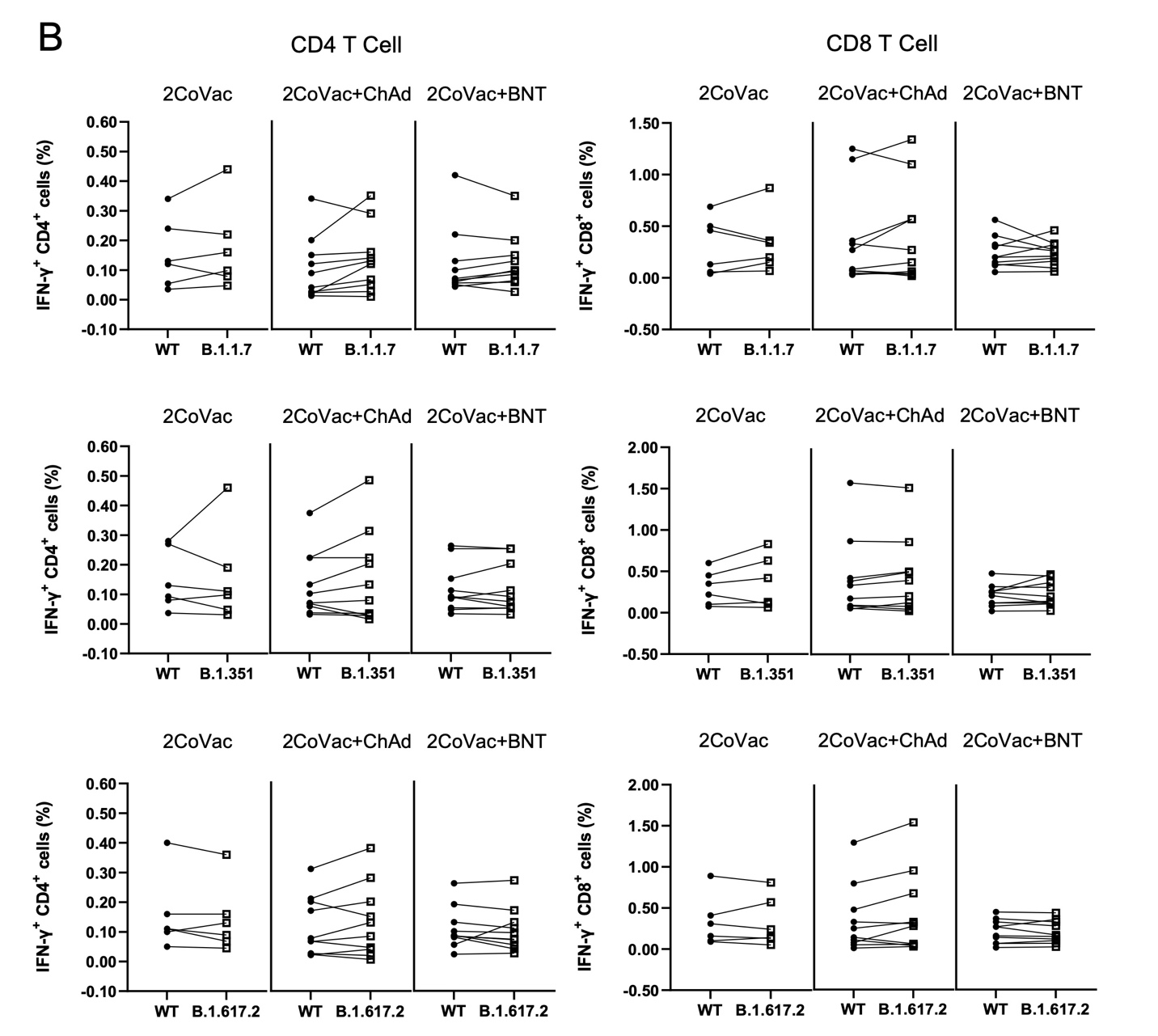


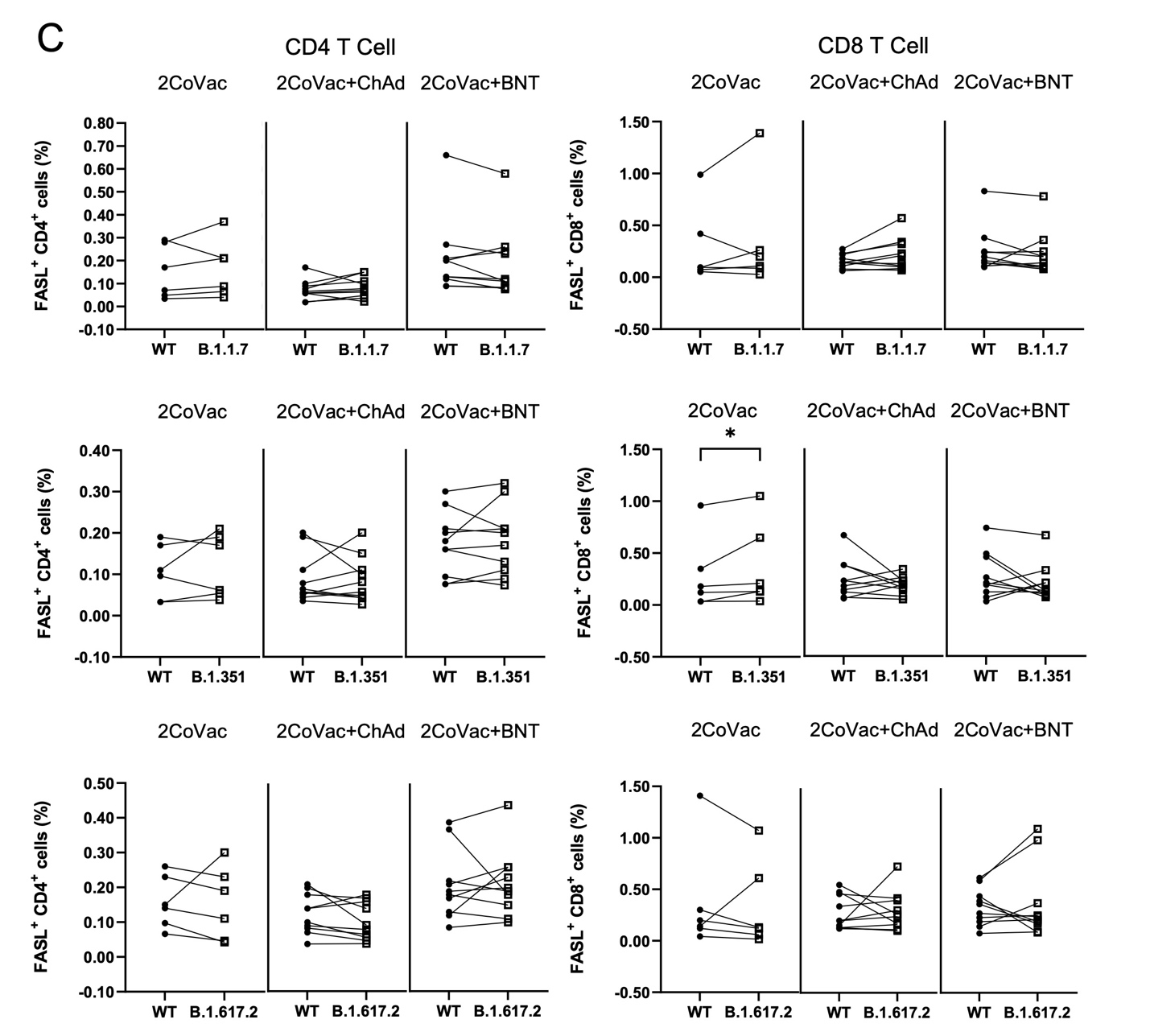


**Figure S3. Comparison of CD4 and CD8 T-cell responses to wild-type spike peptides homology with the mutated regions of spike variants B.1.1.7, B.1.351 and B.1.617.2.** PBMCs were stimulated with spike peptide pools consisting of B.1.1.7 (Alpha), B.1.351 (Beta), or B.1.617.2 (Delta) mutants and their homologous WT peptides. Immunofluorescence staining and flow cytometry were used to determine the frequency of CD4 or CD8 T cells expressing **(A)** IL-17A, **(B)** IFN-γ, and **(C)** FasL. The assays were evaluated before (2CoVac) (N=6) and after boosting with ChAdOx-1 (2CoVac+ChAd) (N=10) or BNT162b2 (2CoVac+BNT) (N=10). The Wilcoxon matched pairs signed-ranks test was used for comparison. ns indicates no significant difference (p > 0.05), and * represents p ≤ 0.05.
